# The Oleic/Linoleic Acid Ratio in Olive (*Olea europaea* L.) Fruit Mesocarp Is Mainly Controlled by *OeFAD2-2* and *OeFAD2-5* Genes Together With the Different Specificity of Extraplastidial Acyltransferase Enzymes

**DOI:** 10.3389/fpls.2021.653997

**Published:** 2021-03-08

**Authors:** M. Luisa Hernández, M. Dolores Sicardo, Angjelina Belaj, José M. Martínez-Rivas

**Affiliations:** ^1^Instituto de la Grasa (IG-CSIC), Campus Universidad Pablo de Olavide, Seville, Spain; ^2^IFAPA Centro Alameda del Obispo, Córdoba, Spain

**Keywords:** *Olea europaea* L., olive, linoleic acid, oleic acid, fatty acid desaturase, gene expression, lipid analysis

## Abstract

Fatty acid composition of olive oil has an important effect on the oil quality to such an extent that oils with a high oleic and low linoleic acid contents are preferable from a nutritional and technological point of view. In the present work, we have first studied the diversity of the fatty acid composition in a set of eighty-nine olive cultivars from the Worldwide Olive Germplasm Bank of IFAPA Cordoba (WOGBC-IFAPA), and in a core collection (Core-36), which includes 28 olive cultivars from the previously mentioned set. Our results indicate that oleic and linoleic acid contents displayed the highest degree of variability of the different fatty acids present in the olive oil of the 89 cultivars under study. In addition, the independent study of the Core-36 revealed two olive cultivars, Klon-14 and Abou Kanani, with extremely low and high linoleic acid contents, respectively. Subsequently, these two cultivars were used to investigate the specific contribution of different fatty acid desaturases to the linoleic acid content of mesocarp tissue during olive fruit development and ripening. Fatty acid desaturase gene expression levels, together with lipid analysis, suggest that not only *OeFAD2-2* and *OeFAD2-5* but also the different specificities of extraplastidial acyltransferase enzymes are responsible for the variability of the oleic/linoleic acid ratio in olive cultivars. All this information allows for an advancement in the knowledge of the linoleic acid biosynthesis in different olive cultivars, which can impact olive breeding programs to improve olive oil quality.

## Introduction

*Olea europaea* L. was one of the first plants to be cultivated for oil production. The olive tree is one of the most important and widespread fruit trees in the Mediterranean basin and, nowadays, is the second most important oil fruit crop cultivated worldwide (Baldoni et al., 2009). Most olive production is destined for olive oil, which is obtained only by mechanical procedures from the olive fruit, with a major contribution of the olive mesocarp to its final composition (Connor and Fereres, 2005). Global olive oil production and consumption have been growing considerably in recent decades thanks to its remarkable organoleptic, technological, and nutritional properties, which are ultimately determined by the metabolites present in the olive oil (Rallo et al., 2018). Among these metabolites, fatty acids that are esterifying triacylglycerols (TAG), which are the major components, are what largely determine the technological and nutritional properties of olive oil (Salas et al., 2000). Oleic acid is the main fatty acid in olive oil and accounts for 55–83% of total fatty acid content. Olive oil also contains variable amounts of linoleic acid (3–21%) and linolenic acid (<1%). The relative content of these fatty acids, which depends mainly on the cultivar, but also on pedoclimatic and management conditions (Beltrán et al., 2004), is an important quality attribute, and it is used to verify the authenticity of olive oil (IOOC, 2001). Noticeably, oleic acid reduces the risk of cardiovascular diseases (Sales-Campos et al., 2013) and suppresses tumorigenesis of inflammatory diseases (Yamaki et al., 2005). Conversely, the excessive linoleic acid intake, due to the high proportion of seed oils in the diet, is associated with a higher risk of hypertension and cardiovascular and carcinogenic diseases (Vos, 2003). Concerning the technological properties, the autoxidative stability of oleic acid is 10-fold higher than that of linoleic acid (O’Keefe et al., 1993). Therefore, olive oils with high oleic and low linoleic acid content are better from a nutritional and technological point of view. Accordingly, the generation of new olive cultivars producing oils with high oleic/linoleic acid ratio is a priority in olive breeding programs.

In higher plants, *de novo* fatty acid synthesis starts in the plastid, yielding primarily palmitoyl-ACP and stearoyl-ACP (Harwood, 2005). Still in the plastid, stearoyl-ACP is desaturated by a soluble stearoyl-ACP desaturase (SAD) to produce oleoyl-ACP, which is the main product of the plastidial fatty acid biosynthesis. These fatty acids are then incorporated into the two glycerolipids pathways that exist in plants: the prokaryotic and eukaryotic pathways. In “18:3 plants”, such as olive, phosphatidylglycerol is the only glycerolipid synthesized in the plastid by the prokaryotic pathway, while the rest of glycerolipids are synthesized in the endoplasmic reticulum (ER) or the plastid using a diacylglycerol (DAG) backbone synthesized in the ER, like the case of galactolipids (Browse and Somerville, 1991). Storage lipids are synthesized in the ER in oil-accumulating tissues via the Kennedy pathway (Gunstone et al., 2007), as well as alternative acyl-CoA-independent mechanism, such as those involving phospholipid:diacylglycerol acyltransferase (PDAT) (Dahlqvist et al., 2000) or diacylglycerol:diacylglycerol transacylase (DGTA) activities (Stobart et al., 1997). In both glycerolipids pathways, desaturation of fatty acids is performed by a series of integral membrane enzymes called fatty acid desaturases. Two sets of ω6 and ω3 membrane-bound fatty acid desaturases have been described, which differ in their cellular localization, lipid substrates, and electron donor system (Shanklin and Cahoon, 1998). The plastidial ω6 and ω3 desaturases (FAD6 and FAD7/8, respectively) are located in the chloroplast, while the microsomal ω6 and ω3 desaturases (FAD2 and FAD3, respectively) are localized in the ER. In the case of ER fatty acid desaturases, two pathways have been reported for the reversible entry of unsaturated fatty acids in phosphatidylcholine (PC) as lipid substrate for further desaturation: an acyl-editing mechanism involving the acyl-CoA pool and the lysophosphastidylcholine acyltransferase (LPCAT) enzyme; and the synthesis of PC from DAG catalysed by the CDP choline:DAG choline phosphotransferase (CPT) and/or PC:DAG choline phosphotransferase (PDCT) enzymes (Bates et al., 2009).

In olive, four genes encoding the soluble SAD have been described: *OeSAD1* (Haralampidis et al., 1998), *OeSAD2*, *OeSAD3* (Parvini et al., 2016), and *OeSAD4* (Contreras et al., 2020). Regarding membrane bound desaturases, five genes encoding microsomal oleate desaturases (*OeFAD2-1* to *OeFAD2-5*) have been isolated and characterized by Hernández et al., 2005, 2009, 2020, whereas only one *OeFAD6* gene has been described so far (Banilas et al., 2005; Hernández et al., 2011). Additionally, four members of the olive linoleate desaturase gene family have been characterized, two microsomal (*OeFAD3A*, Banilas et al., 2007; *OeFAD3B*, Hernández et al., 2016) and two plastidial (*OeFAD7-1*, Poghosyan et al., 1999; *OeFAD7-2*, Hernández et al., 2016).

In previous studies, we have characterized the olive fatty acid desaturase genes involved in the unsaturated fatty acid composition of olive oil in the two main cultivars for olive oil production such as Picual and Arbequina (Hernández et al., 2005, 2009, 2016, 2020; Parvini et al., 2016). However, the genetic control of its variability among olive cultivars is still poorly understood. For this reason, we extended the study to the cultivars of a previously developed core collection (Core-36) with a wide genetic diversity (Belaj et al., 2012), which allowed the identification of two olive cultivars with highly contrasting linoleic acid content: Klon-14 and Abou Kanani. These two cultivars were further used to determine the expression levels of genes encoding fatty acid desaturases in their mesocarp tissues during olive fruit development and ripening, in order to investigate their involvement in the regulation of the oleic and linoleic acid content. In addition, lipid analysis has been carried out to examine the possible implication of other genes and enzymes of the lipid biosynthetic pathway. Our data shed light on the understanding of the transcriptional and metabolic mechanisms that control the oleic/linoleic acid ratio in olive cultivars, to improve olive oil quality.

## Materials and Methods

### Plant Material

A total of 89 cultivars from the Worldwide Olive Germplasm Bank of Cordoba (WOGBC-IFAPA) located at the Andalusian Institute of Agricultural and Fisheries Research and Training (IFAPA), “Alameda del Obispo,” in Cordoba (Spain) were included in this work (Supplementary Table 1). The study was also extended to the 36 cultivars (Supplementary Table 2) corresponding to a previously established core collection (Core-36) (Belaj et al., 2012), which included 28 cultivars from the 89 selected from the WOGBC. Trees, two per accession, were cultivated in the same agroclimatic conditions at the experimental orchards of IFAPA (Alameda del Obispo, Córdoba, Spain) at 6 m × 5 m spacing, using standard culture practices (Gómez-Gálvez et al., 2020). In order to obtain representative samples of the olive fruits from all parts of the olive trees, fruits were harvested by hand all around from two trees per cultivar, mixed and spliced into three different pools, that constitute three different biological replicate, for oil fatty acid analysis and mesocarp developmental studies.

For olive oil extraction, fruit samples from the 89 cultivars from the WOGBC-IFAPA were harvested during the seasons 2008/2009 and 2009/2010, while samples from the Core-36 were harvested during season 2011/2012. The harvest was carried out at the turning stage (28–31 weeks after flowering [WAF]), and oil was extracted consecutively.

In the case of developmental studies, mesocarp tissue were harvested from the selected cultivars, Klon-14 and Abou Kanani, at different WAF corresponding to different developmental stages of the olive fruit: green (20 WAF); yellowish (24 WAF); turning or veraison (28 WAF) and purple or mature (31 WAF). Immediately after harvesting, olive mesocarp was frozen in liquid nitrogen and stored at −80°C until its use.

### Olive Oil Extraction

Oil was extracted from olive fruits using a laboratory oil extraction plant (Abencor, Comercial Abengoa, SA, Seville, Spain) that mimics the industrial virgin olive oil (VOO) production at a laboratory scale according to Martínez et al. (1975). Fruit crushing was carried out with a stainless steel hammer mill working at 3000 rpm and a 5 mm sieve. The Abencor thermo-beater was used for the malaxation step for 30 min at 28°C. Finally, paste centrifugation was performed in a basket centrifuge for 1 min at 3500 rpm. Oils were decanted, filtered through paper, and stored under a nitrogen atmosphere at −20°C until analysis.

### Lipid and Fatty Acid Analysis

Olive fruit mesocarp tissue was heated at 70°C for 30 min with isopropanol to inactivate endogenous lipase activity. Lipids were extracted as described by Hara and Radin (1978) and lipid separation was carried out by thin layer chromatography (Hernández et al., 2008).

Fatty acid methyl esters of the different olive oil extracted and the lipid preparations obtained from the olive mesocarp at different stages of development were produced by acid-catalyzed transmethylation (Garcés and Mancha, 1993) and analyzed by gas chromatography (Román et al., 2015). Heptadecanoic acid was used as internal standard to calculate the lipid and fatty acid content in the samples. Results are expressed either in mol percent of the different fatty acids or in μg of fatty acid per mg of FW and are presented as means ± SD of three biological replicates.

### Total RNA Isolation and cDNA Synthesis

Total RNA isolation was performed as described by Hernández et al. (2005) using 1.5 g of frozen olive mesocarp. RNA quality verification, removal of contaminating DNA, and cDNA synthesis were carried out according to Hernández et al. (2009).

### Quantitative Real-Time PCR

The expression levels of the olive fatty acid desaturase genes were determined by quantitative real-time PCR (qRT-PCR) using a CFX Connect real-time PCR System and iTaq Universal SYBR Green Supermix (BioRad, California, United States) as previously described (Hernández et al., 2019). Primers for gene-specific amplification (Supplementary Table 3) were previously described for *OeSAD* genes (Parvini et al., 2016), for *OeFAD2* and *OeFAD6* genes (Hernández et al., 2009, 2020), and *OeFAD3* and *OeFAD7* genes (Hernández et al., 2016). The housekeeping olive ubiquitin2 gene (*OeUBQ2* and AF429430) was used as an endogenous reference to normalize (Hernández et al., 2009). The relative expression level of each gene was calculated using the equation 2^−Δ*Ct*^ where ΔCt = (Ct_GOI_ – Ct_UBQ__2_) (Livak and Schmittgen, 2001; Pfaffl, 2004). This method gave us the advantage to make comparisons in the level of gene expression across developmental stages, cultivars, and genes. The data are presented as means ± SD of three biological replicates, each having two technical replicates per 96-well plate.

## Results and Discussion

### Variability of Oleic and Linoleic Acid Content in an Olive Cultivar Core Collection (Core-36)

**FIGURE 1:**
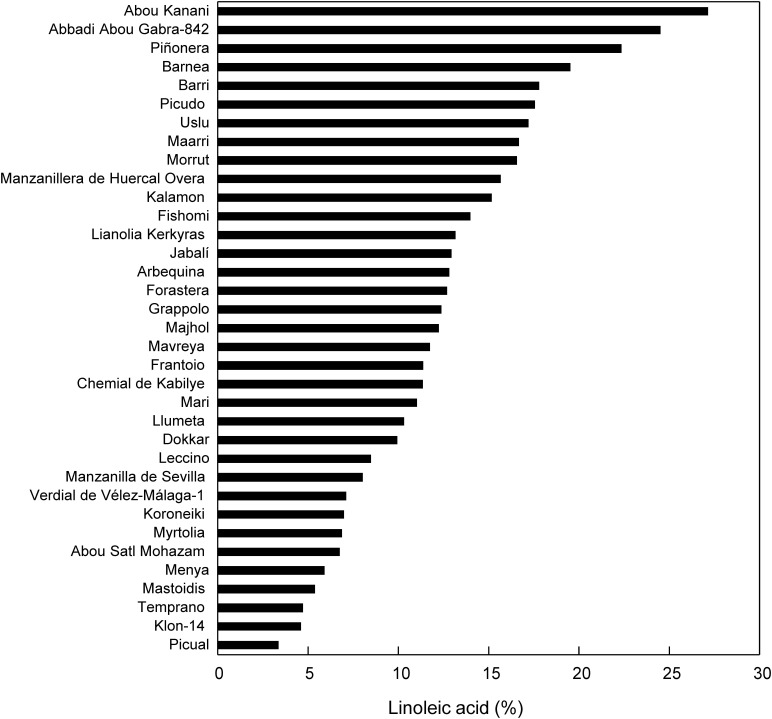
Linoleic acid percentage in the oils of the Core-36. Olive fruits were hand-harvested at the turning stage during the season 2011/2012. Oil extraction and fatty acid analysis were determined as described in materials and methods. Data represent the mean of three biological replicates. In all the cases SD < 3% of mean values.

In accordance with previous studies in olive (León et al., 2018), the fatty acid analysis of 89 olive cultivars showed a high level of diversity conserved in the WOBGC-IFAPA located in Córdoba (Spain). As expected, oleic acid was the most abundant fatty acid, followed by palmitic and linoleic acids (Supplementary Table 1). This variability could be explained only by the genetic component, since the culture conditions, fruit ripeness, and oil extraction conditions were fixed, further confirming that the genotype is the major source of variability for olive oil fatty acid composition (Ripa et al., 2008). To further explore the diversity in the oleic/linoleic acid ratio in different olive cultivars, we decided to analyze the fatty acid composition of a Core-36, which holds most of the genetic diversity found in the WOGBC-IFAPA (Belaj et al., 2012) (Supplementary Table 2). Thus, among the most abundant fatty acids, the highest degree of variability in the fatty acid profile was found for the oleic and linoleic acid percentage in the oils of the Core-36, in such a way that the coefficient of variation for the oleic/linoleic acid ratio was 66.48% (Supplementary Table 4). The mean percentages of oleic and linoleic acids were 66.84 and 12.35%, respectively, being these values very similar to the ones found in cultivated material (León et al., 2018). The variability intervals of oleic and linoleic acids ranged from 46.24–79.15% and 3.34–27.12%, respectively, being the cultivars Picual and Abou Kanani the ones with the most contrasting linoleic acid percentage (Figure 1). Interestingly, these variability ranges were slightly lower than those observed for oleic and linoleic acids in the segregating progeny of the cross between Picual and Arbequina cultivars (50.6–81.9% and 2.9–23.1%, respectively) (Hernández et al., 2017). In addition, the oleic/linoleic acid ratio was inversely proportional to the linoleic acid content (Supplementary Table 2), clearly indicating that the variability of linoleic acid content found in the Core-36 oils is mainly caused by different varietal capacities of oleate desaturase enzymes. High variability was also found between the saturated/unsaturated ratio, mainly due to the differences found in palmitic acid content, with the highest (23.70%) and the lowest (9.68%) levels detected in cultivars Dokkar and Majhol-152, respectively (Supplementary Table 2). A high level of variability of the Core-36 has also been previously reported for the VOO phenolic and volatile fractions (García-Rodríguez et al., 2017; García-Vico et al., 2017), and tocopherol composition (Pérez et al., 2019). Therefore, our results confirm the evidence that cultivar differences in the fatty acid composition of olive oils are due to the genetic component (Tous et al., 2005; De la Rosa et al., 2013; Montaño et al., 2016). The Core-36 analyzed in this study facilitates the evaluation and utilization of its genetic diversity to understand the mechanism underlying fatty acid desaturation in olive for crop improvement.

### *OeFAD2-2* and *OeFAD2-5* Are the Main Genes Controlling the Oleic/Linoleic Acid Ratio in Olive Mesocarp

Previously, we have investigated the specific contribution of each olive oleate desaturase gene to the linoleic acid content in the olive oil using “Picual” and “Arbequina,” because these are the two main cultivars for olive oil production (Hernández et al., 2009, 2016, 2020; Parvini et al., 2016). In this work, we decided to select two olive cultivars with high contrasting amounts of linoleic acid, with the aim of investigating the genotypic differences in olive mesocarp fatty acid desaturation. Specifically, we chose cultivars Klon-14, with a low linoleic acid content (4.58%), and Abou Kanani, with high linoleic acid levels (27.12%) (Figure 1) for a comparative molecular and metabolic analysis during olive fruit development and ripening.

**FIGURE 2:**
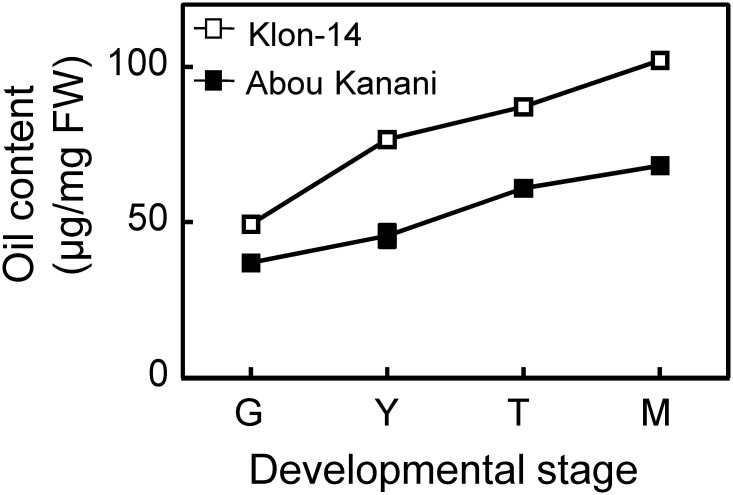
Oil content in the mesocarp tissue of Klon-14 and Abou Kanani cultivars at different stages of development. Lipid content was determined as described in materials and methods. Data are presented as means ± SD of three biological replicates. G, Green; Y, yellowish; T, turning; M, matured.

**FIGURE 3:**
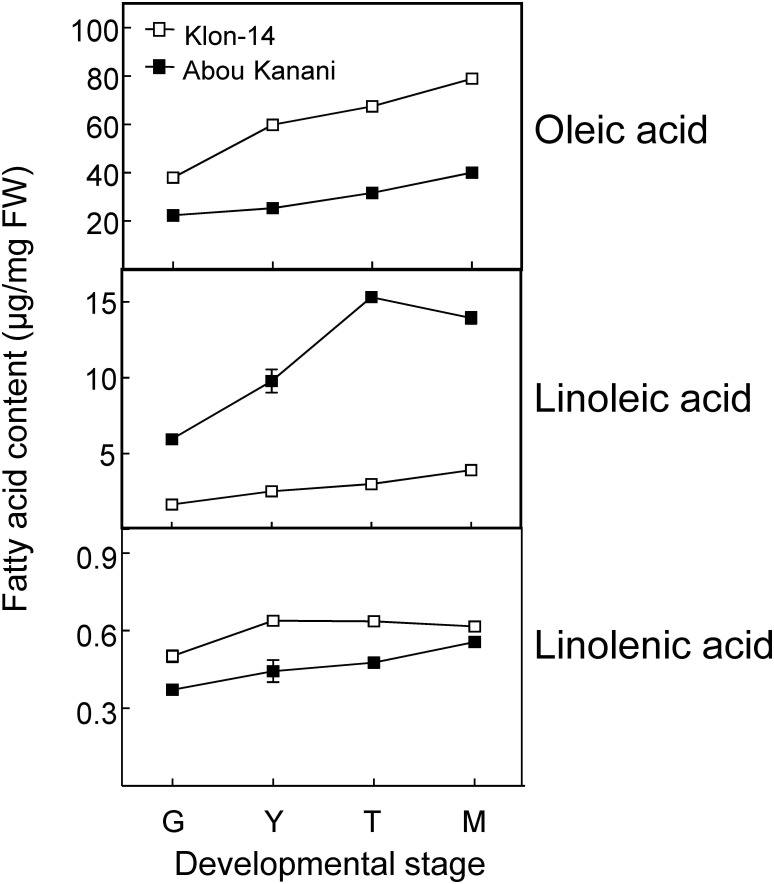
Oleic, linoleic, and linolenic acid contents in the mesocarp tissue of Klon-14 and Abou Kanani cultivars at different stages of development. Fatty acid content was determined as described in materials and methods. Data are presented as means ± SD of three biological replicates. G, Green; Y, yellowish; T, turning; M, matured.

As shown in Figure 2, oil accumulation in olive mesocarp during development and ripening increased in both cultivars studied, in parallel to the oleic, linoleic, and linolenic acids content (Figure 3). Besides, the amount of oleic acid in “Klon-14” mesocarp was much higher than in “Abou Kanani,” reaching up to 80 μg/mg FW at the matured stage. In contrast, linoleic acid levels increased moderately in “Klon-14” mesocarp during the olive fruit development and ripening, while in “Abou Kanani” they increased abruptly during the developing period, reaching a maximum at the turning stage. These patterns of linoleic acid accumulation observed in Klon-14 and Abou Kanani cultivars were respectively similar, to those reported for “Picual,” “Koroneiki” and “Mari,” and for “Arbequina” and “Shengeh” (Hernández et al., 2009; Parvini et al., 2015). Regarding linolenic acid, its content was considerably low in both cultivars studied, although slightly higher in “Klon-14” mesocarp. This result was different from those reported by Hernández et al. (2016), in which “Arbequina,” the cultivar with a higher linoleic acid content, also showed higher level of linolenic acid, particularly during the olive fruit developing period.

**FIGURE 4:**
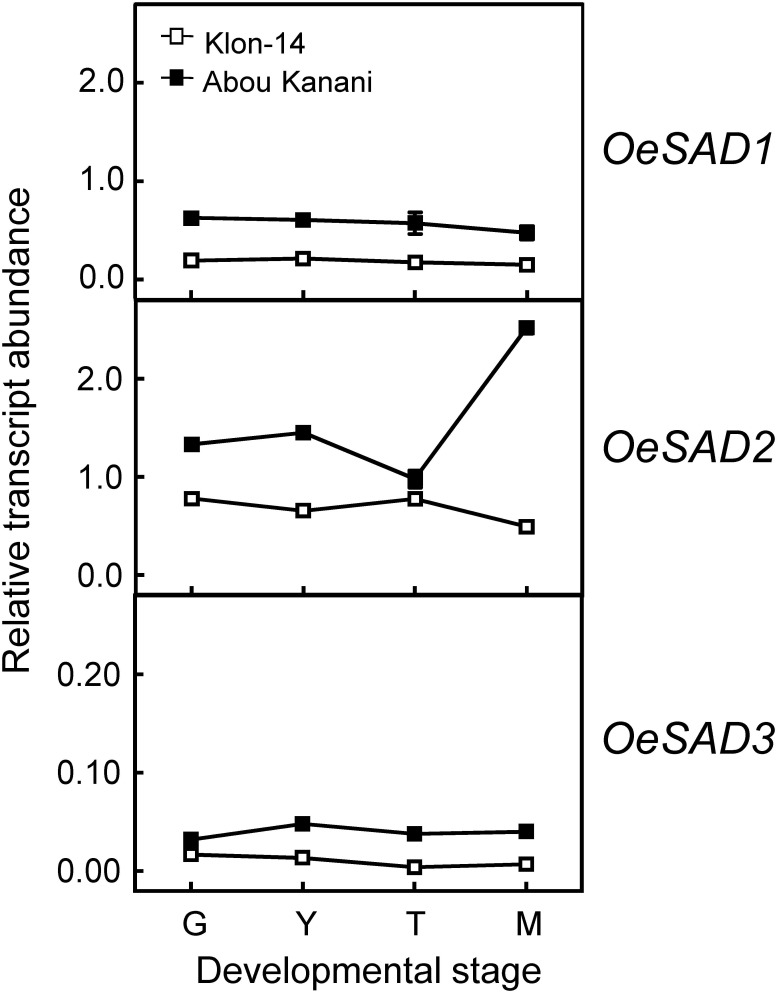
Relative transcript abundance of olive *SAD* genes in the mesocarp tissue of Klon-14 and Abou Kanani cultivars at different stages of development. The relative transcript abundance was determined by qRT-PCR as described under materials and methods. Data are presented as means ± SD of three biological replicates. G, Green; Y, yellowish; T, turning; M, matured.

Subsequently, we determined the expression levels of olive fatty acid desaturase genes responsible for the biosynthesis of oleic (*OeSAD*), linoleic (*OeFAD2* and *OeFAD6*), and linolenic acid (*OeFAD3* and *OeFAD7*). The relative transcript abundance analysis of olive *SAD* genes showed that *OeSAD2* gene expression levels were the highest throughout the olive mesocarp development and ripening in both cultivars studied (Figure 4). These data point to the *OeSAD2* gene as the main candidate for oleic acid production in the olive mesocarp. In contrast, *OeSAD1*, and especially *OeSAD3*, exhibited lower expression levels compared to *OeSAD2*. These data are in agreement with previous studies in other olive cultivars (Parvini et al., 2016; Contreras et al., 2020), further confirming that *OeSAD2* is the main gene that contributes to the oleic acid synthesis in the olive mesocarp and, therefore, to its content in the olive oil.

The higher *OeSAD2* gene expression levels observed in “Abou Kanani” compared to “Klon-14” mesocarp (Figure 4) did not correlate with the total oil content, which was higher in Klon-14 cultivar (Figure 2). These results are consistent with those reported by Parvini et al. (2016) in Picual and Arbequina cultivars, indicating that in olive, the *SAD2* gene, which seems to be mainly responsible for oleic acid synthesis, was not directly associated with TAG accumulation. In contrast, a full correlation between expression levels of a *SAD* gene and oil accumulation has been reported in many plant species (Cummins et al., 1993; Liu et al., 2009; Shilman et al., 2011; Li et al., 2014; Kilaru et al., 2015).

On the other hand, Parvini et al. (2015) proposed that the higher oleic/linoleic ratio in Mari mesocarp, in comparison to Shengeh, was due to the higher expression levels of *OeSAD2* gene, together with lower *OeFAD2-2* expression levels. However, this hypothesis was discarded in a later study by Parvini et al. (2016), who observed the highest *OeSAD2* expression level in Arbequina and Manzanilla cultivars, which are characterized by low and high oleic/linoleic acid ratio, respectively. The results obtained in the present study agree with Parvini et al. (2016) since “Abou Kanani” mesocarp, which showed higher *OeSAD2* expression levels (Figure 4), exhibit a lower oleic/linoleic acid ratio (Supplementary Table 2), supporting the hypothesis that olive *SAD2* gene is not primarily involved in the control of the linoleic acid content in the olive mesocarp.

**FIGURE 5:**
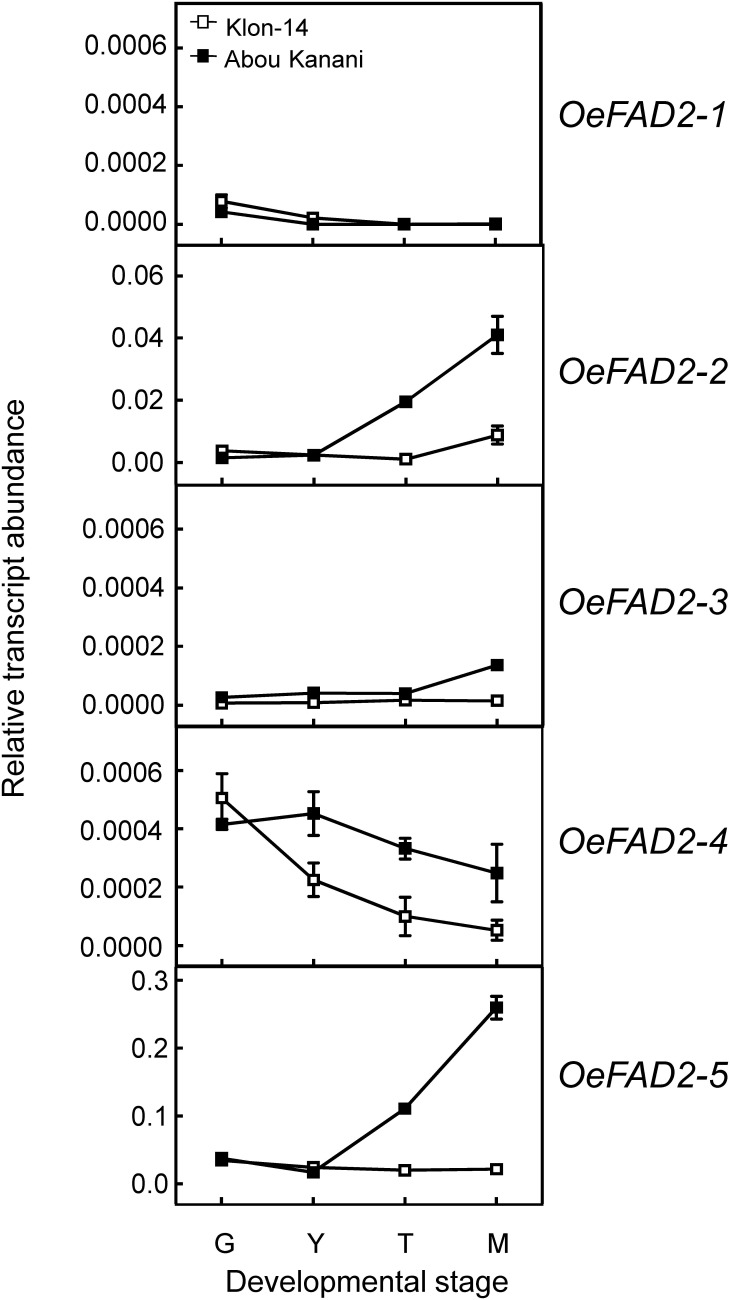
Relative transcript abundance of olive *FAD2* genes in the mesocarp tissue from Klon-14 and Abou Kanani cultivars at different stages of development. The relative transcript abundance was determined by qRT-PCR as described under materials and methods. Data are presented as means ± SD of three biological replicates. G, Green; Y, yellowish; T, turning; M, matured.

**FIGURE 6:**
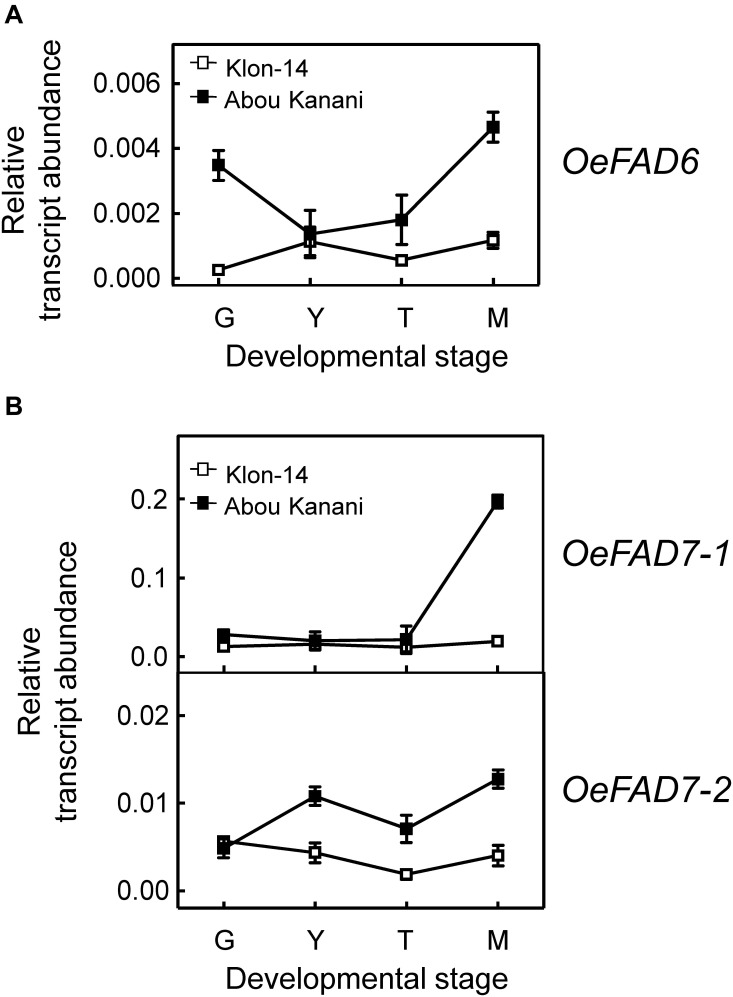
Relative transcript abundance of olive *FAD6* **(A)** and *FAD7* **(B)** genes in the mesocarp tissue from Klon-14 and Abou Kanani cultivars at different stages of development. The relative transcript abundance was determined by qRT-PCR as described under materials and methods. Data are presented as means ± SD of three biological replicates. G, Green; Y, yellowish; T, turning; M, matured.

Regarding oleate desaturase genes (*FAD2* and *FAD6*), *OeFAD2-2* and *OeFAD2-5* showed the highest expression levels in Klon-14 and Abou Kanani cultivars (Figures 5, 6A). In addition, we could observe a correlation between the *OeFAD2-2* and *OeFAD2-5* gene expression levels (Figure 5) and the linoleic acid content (Figure 3) in “Klon-14” and “Abou Kanani” mesocarp, particularly during the ripening period. Furthermore, the higher transcript levels detected for these two *FAD2* genes in “Abou Kanani” mesocarp compared with “Klon-14” (Figure 5) agree with the high linoleic acid content observed for the mesocarp tissue from “Abou Kanani” relative to that of “Klon-14” (Figure 3). All these results indicate that *OeFAD2-2* and *OeFAD2-5* are the main genes responsible for the linoleic acid synthesis in the olive mesocarp and, therefore, for the linoleic acid content of olive oil. This conclusion is in keeping with that described for other olive cultivars, either in the case of *OeFAD2-2* (Hernández et al., 2009; Parvini et al., 2015; Contreras et al., 2020), or *OeFAD2-5* (Hernández et al., 2020). The fact that the mesocarp tissue possesses two genes involved in the oleic acid desaturation may be to assure the linoleic acid biosynthesis all over the prolonged developmental period of olive fruit that takes approximately 35–40 weeks (Hernández et al., 2020).

Our whole data strongly support the hypothesis that the linoleic acid content is essentially determined by the olive cultivar, which is ultimately controlled by the *OeFAD2-2* and *OeFAD2-5* gene expression levels. Additionally, these results point to *OeFAD2-2* and *OeFAD2-5* as good candidate genes for the co-localized QTLs for oleic and linoleic acids, as well as for monounsaturated and polyunsaturated fatty acids, and the oleic/linoleic acid ratio, identified in a linkage map of a “Picual” × “Arbequina” progeny (Hernández et al., 2017). This information could be particularly useful for the development of molecular markers for the high oleic/low linoleic character, to increase the efficiency of breeding programs for the selection of new and improved olive cultivars.

Concerning linoleate desaturases, the analysis of their gene expression levels during “Klon-14” and “Abou Kanani” mesocarp development and ripening revealed that *OeFAD3A* and *OeFAD3B* gene transcripts were undetectable at the stages studied, while *OeFAD7-1* and *OeFAD7-2* increased all along the development and ripening period, particularly in “Abou Kanani” mesocarp (Figure 6B). Analogous expression patterns for olive *FAD3* and *FAD7* genes have been reported before for the mesocarp tissue of “Koroneiki” (Poghosyan et al., 1999; Banilas et al., 2007), “Canino,” “Frantoio,” and “Moraiolo” (Matteuci et al., 2011), and “Picual” and “Arbequina” (Hernández et al., 2016). These data indicate that *OeFAD7*, and not *OeFAD3*, could be the main genes responsible for the linolenic acid content in the mesocarp and, therefore, in the olive oil. In fact, Hernández et al. (2016) postulated that in olive mesocarp, the linoleic acid synthesized in the ER by *OeFAD2* genes is transferred to the chloroplast where *OeFAD7-1* and *OeFAD7-2* genes are responsible for its desaturation to produce linolenic acid, which is then exported again to the ER to be incorporated into TAG. Alternatively, olive FAD7 enzymes can act on ER lipids while located in chloroplast as it has been proposed for FAD7 in *Chlamydomonas reinhardtii* (Nguyen et al., 2013). Although the specific mechanism by which trafficking of fatty acids out of and into the chloroplast has not yet been elucidated, the most recent advances suggest that lipid transport is achieved by multiple mechanisms which include membrane contact sites with specialized protein machinery, such as TGD complexes (LaBrant et al., 2018). It is interesting to point out the higher expression levels of *OeFAD7* genes observed in “Abou Kanani” mesocarp compared to “Klon-14” (Figure 6A), with “Abou Kanani” exhibiting lower levels of linolenic acid (Figure 3). Similar results were reported in Picual and Arbequina cultivars, where the higher *OeFAD7* genes expression levels detected in “Picual” mesocarp did not correspond with a higher amount of linolenic acid. One possible explanation could be that the linoleate desaturation in olive mesocarp may be subjected to post-transcriptional regulation. In soybean cellular cultures, *FAD7* regulation by light is carried out by post-translational regulatory mechanisms (Collados et al., 2006). On the other hand, the explanation may lie in the mechanism involved in the transport of fatty acids across the chloroplast membrane, which remains to be completely elucidated.

### Cultivar Differences in LPCAT Specificity Could Play a Significant Role in Determining the Linoleic Acid Content of TAG Molecules

**FIGURE 7:**
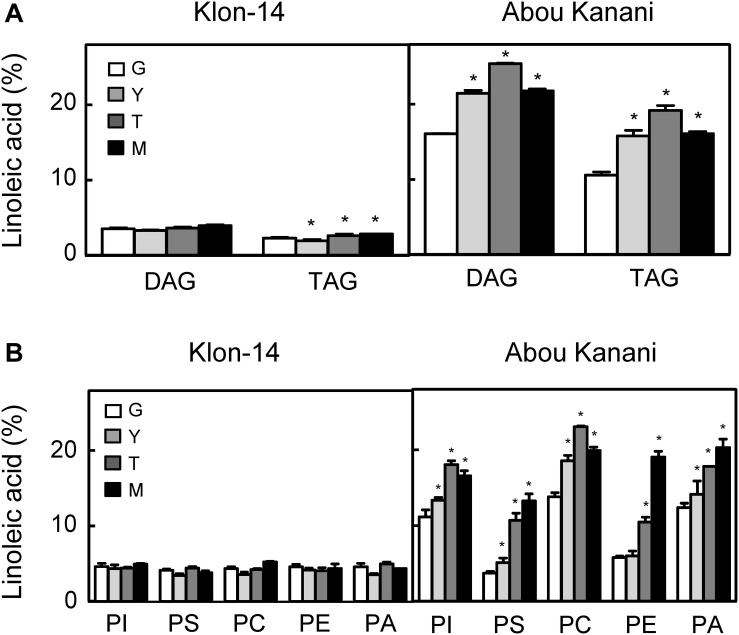
Linoleic acid percentage in neutral lipids **(A)** and phospholipids **(B)** from “Klon-14” and “Abou Kanani” mesocarp tissue at different stages of development. The linoleic acid percentage was determined as described under materials and methods. Data are presented as means ± SD of three biological replicates. G, Green; Y, yellowish; T, turning; M, matured. *Indicates significantly different (*P* < 0.05) to G stage by two-way ANOVA with a Bonferroni *post hoc* test.

With the aim of further investigation of the metabolic pathways for the biosynthesis and accumulation of linoleic acid in olive mesocarp, we analyzed the fatty acid composition in different lipid classes from “Klon-14” and “Abou Kanani” mesocarp at different stages of olive fruit development and ripening. The linoleic acid percentage in DAG and TAG was considerably higher in “Abou Kanani” than in “Klon-14” mesocarp (Figure 7A), in agreement with the high linoleic acid levels found in “Abou Kanani” oil (Figure 1). In addition, while the increase of linoleic acid in neutral lipids in “Klon-14” mesocarp was slow and gradual during the olive fruit development and ripening, in “Abou Kanani” mesocarp the linoleic acid increased sharply from the beginning of fruit development in both, DAG, and TAG (Figure 7A). These results correlate quite well with *OeFAD2-2* and *OeFAD2-5* genes expression levels in both cultivars (Figure 5), further confirming the role of these two genes in the production of linoleic acid in the olive mesocarp.

Furthermore, the analysis of linoleic acid content in phospholipids revealed clear differences between both cultivars (Figure 7B). Interestingly, the percentage of linoleic acid was low (∼4%) and did not change significantly in any of the phospholipids studied in “Klon-14” mesocarp during development and ripening. In contrast, in “Abou Kanani” mesocarp, the linoleic acid increased considerably during the olive fruit development and ripening, reaching up to 20% of total fatty acid, particularly in PC, which showed the highest values (Figure 7B). This increase in PC linoleic acid levels was accompanied by a reduction in oleic acid (Supplementary Table 5), indicating a high rate of oleate desaturation in this cultivar.

The parallel tendency detected in the linoleic acid content in PC and PA, unchanged in “Klon-14” and increased in “Abou Kanani,” points to the acyl-editing mechanism catalyzed by LPCAT and further incorporation to the Kennedy pathway as the main entry route of linoleic acid synthesized in PC into *de novo* DAG, versus the CPT and/or PDCT pathway that produce PC-derived DAG as substrate for TAG synthesis (Bates, 2016). The observation that the linoleic acid metabolism differs in different olive cultivars has been previously reported by Hernández et al. (2020) in “Picual” and “Arbequina.” Interestingly, in all cultivars studied so far, a similar trend in linoleic acid content in PC and PA has been observed (Figure 7B; Hernández et al., 2020), highlighting the role of LPCAT in olive mesocarp lipid metabolism.

On the other hand, it is likely that in the case of “Abou Kanani” mesocarp, the high degree of desaturation of oleic to linoleic acid allows the accumulation of this fatty acid in all lipid classes, including TAG. However, in “Klon-14” mesocarp it seems that the scenario is different. The observation that the linoleic acid slightly increased in TAG while did not change in phospholipids (Figure 7) indicates that the small amount of linoleic acid that is synthesized in PC, due to the low *OeFAD2-2* and *OeFAD2-5* genes expression levels (Figure 5), is incorporated into TAG. Differently, in “Picual” and “Arbequina” mesocarp, a distinct preference of DGAT enzyme for linoleoyl-CoA has been proposed to explain the accumulation of this fatty acid in phospholipids or TAG, respectively (Hernández et al., 2020).

However, the most striking observation was that the oleic acid content decreased in PC in “Klon-14” mesocarp, while there is an increase in palmitic and stearic acids (Supplementary Table 6). These results could be explained by a decrease in oleic acid production due to the low *SAD2* expression levels detected in this cultivar (Figure 4). However, this hypothesis is not plausible since the total oleic acid content increased in “Klon-14” mesocarp during the olive fruit development and ripening (Figure 3). An alternative explanation could be a reduced incorporation of oleic acid into PC. This phospholipid is the major site of acyl-editing, and LPCAT enzyme is responsible for the partition of newly synthesized fatty acid between the acyl-CoA pool and PC. Therefore, LPCAT plays a major role in supplying oleate to the PC pool for desaturation (Wang et al., 2012). Although the substrate specificity of the acyltransferases of the Kennedy pathway is one of the determining factors of TAG composition (Ohlrogge and Browse, 1995; Jeppson et al., 2019), Menard et al. (2018) reported that natural variation in a gene encoding the acyl-editing enzyme LPCAT influences TAG composition in Arabidopsis seed. Therefore, the low *OeFAD2-2* and *OeFAD-5* genes expression levels, together with a decreased incorporation of oleic acid into PC by LPCAT, could explain the low linoleic acid levels found in “Klon-14” oil.

## Conclusion

The oleic and linoleic acid contents exhibit the highest degree of variability in the fatty acid composition of olive oils from the Core-36 olive cultivar collection. Gene expression data in olive mesocarp during development and ripening from two olive cultivars with contrasting amounts of linoleic acid suggest that *OeFAD2-2* and *OeFAD2-5* genes play a key role in the variability of the oleic/linoleic acid ratio in olive cultivars. In addition, the different fatty acid profile of individual lipids in both cultivars suggests that differences in the specifities of extraplastidial acyltransferase enzymes could be also involved in the control of the oleic/linoleic acid ratio in olive. The present study highlights the importance of cultivar collections not only for the selection of optimal parents for breeding programs but also as a key source of plant material to carry out basic research. Finally, all this information will allow the development of molecular markers to be used in the marker-assisted selection of new olive cultivars that produce oils with high oleic and low linoleic contents, increasing the efficiency of olive breeding programs to obtain VOO with improved quality.

## Data Availability Statement

The original contributions presented in the study are included in the article/Supplementary Material, further inquiries can be directed to the corresponding author/s.

## Author Contributions

MLH managed and performed the harvest of plant material, carried out RNA isolation and cDNA synthesis, analyzed the data, and drafted the manuscript. MDS performed the harvest of plant material and qRT-PCR and lipid analysis. AB contributed to the selection and collection of the plant materials and to the manuscript revision. JM-R conceived and designed the study, analyzed the data, and contributed to manuscript revision. All authors discussed, commented, and approved the final version of the manuscript.

## Conflict of Interest

The authors declare that the research was conducted in the absence of any commercial or financial relationships that could be construed as a potential conflict of interest.
